# Some Remarks on the Dengue Epidemic

**Published:** 1885-11

**Authors:** W. J. Burt

**Affiliations:** Austin, Texas


DANIEL'S

### Medical ★ Journal,

PUBLISHED MONTHLY AT

AUSTIN, TEXAS.

Vol. I.] NOVEMBER, 1885. [No. 5.

"*Scribimus indocti, doctique!*"

### Original Articles.

☞ CONTRIBUTED EXCLUSIVELY TO THIS JOURNAL. ☜

*The Articles in this Department are accepted, and published with the understanding that we are not responsible for, nor do we indorse the views and opinions of the writers, by so doing.*

### SOME REMARKS UPON DENGUE AND THE EPIDEMIC AS IT PREVAILED IN AUSTIN IN AUGUST, SEPTEMBER AND OCTOBER, 1885.

*By W. J. Burt, M. D., Austin, Texas.*

Read Before the Travis County Medical Association, and Ordered to be Published in Daniel's Texas Medical Journal.

DENGUE is known by many names. Probably the most appropriate synonym for it, is "Break Bone Fever." This phrase, at least, carries with it vivid recollections to those who were the recipients of its favor in the recent epidemic.

The term "Dengue," however, in a nosological sense, has been accepted by leading authors on the subject and by the college of physicians and surgeons of London.

HISTORY.

The first authentic records of dengue dates about 1824; or in other words the differential diagnosis of the disease was so confused, that authors gave no clear history of it, previous to that time.

There was a fever in Spain in 1764, which resembled very much the dengue of 1826–28. There was also an epidemic fever in Philadelphia in 1780, described by the celebrated Dr. Rush, and called by him "Bilious Remittent Fever." The history and symptoms were in many points similar to those of the epidemic of dengue of 1828 and 1850. Dr. Dickson, of Charleston, South Carolina, who for accuracy and incisive description, has hardly had an equal, believes that the true "eruptive dengue fever" never prevailed, epidemically, in the coast states until the latter part of the summer of 1828. He, however, says that there was a fever in Savannah, Georgia, in 1826, but he thinks it was a hybrid fever and not true dengue. The dengue of 1828 was imported from the West Indies. After this it seemed to have disappeared on the southern coast until 1848 and 1850 when it prevailed all along the coast, particularly in Charleston, Savannah and Augusta. Then again in 1873 when it prevailed in New Orleans and in many places in Louisiana and Texas. It was in 1873 when the first epidemic of dengue prevailed in Austin, when it spread over the entire city. Dengue again visited Austin last fall and winter, in many localities, but was of a mild type and confined to a few neighborhoods.

The history of the disease, in this and other countries, shows that it has no regular periods of visitation—at times but one or two years between epidemics—then as long as ten, twenty or more years between the invasions.

So we can predicate nothing from the history of dengue in reference to the time another epidemic will visit us.

The truth is we know very little of the laws that govern the course of epidemics or of the nature of the infective matter that enters our bodies and produces such ravages in the blood and organs. While we are ignorant of those laws that control epidemics, it may be said of a number of them, that their infective particles or germs are chiefly dependent for their fertility and extended spread on the presence of filth—or are incubated and fertilized amid putrefactive decomposition of vegetable or animal matters. For instance, we are able to associate malarial fevers, with vegetable decomposition, while typhus fever is more allied, in its etiology, with animal matter and animal excretions. The air, its humidity, its temperature and its electrical conditions are signficant factors in the putrefactive or fermentive process. These infective particles, or germs, or micrococci, may die for want of an opportunity to spring into active work. The precise unity of conditions may not obtain, although the material is in wait.

Or, on the other hand, the turned up soil, the filthy streets and alleys, and the horrible cesspools of almost every household, may produce favorable atmospheric conditions for the development and spread of any imported germs, into a fearful epidemic. It is easy enough now to say that no case of small pox would likely arise without an antecedent case, any more than a child without a parent. But we cannot say this of diphtheria or typhoid fever. But for small pox on the one hand, or diphtheria on the other hand, to become epidemic, the fact of the presence of an infective element or germ is not sufficient; they must also have those favorable conditions and associations around them to fertilize and put in motion their infective germs.

The recent epidemic of "Dengue," whether it originated in Austin de novo or was the result of a general atmospheric or telluric condition, or was imported into the city, it is safe to say was governed in its epidemicity by the same laws and principles that govern other epidemics. While we are yet ignorant of the cause of dengue, we submit that its epidemic work and results, have been in an inverse ratio to the favorable conditions for its propagation. If for instance the first cases of the present epidemic were imported and did not originate in the city, it is fair to conclude from the experience and observation of medical men and from the logic of the facts themselves, that but few, if any cases, except the imported ones, would have occurred, *provided* the conditions favorable to its spread, had not already existed in the city. Now, what that "favorable condition" is, or what factors enter in, to constitute it, is so far an unsolved problem. We recognize the fact but not the factors.—Not many years ago two cases of yellow fever occurred in Austin. *They were imported cases*, no epidemic prevailed. There were a number of cases of "dengue"

in the city last fall and winter, but no general epidemic resulted. Isolated cases of "dengue" have frequently occurred in various parts of the coast states from year to year, when no epidemic prevailed. Therefore, we reason, that for "dengue" to become epidemic, the local conditions favorable to the *fertilization* and *mobilization* of its infectious germs, must co-exist with the case.

### THE CAUSE OF THE EPIDEMIC.

The cause of the recent epidemic, as well as those of 1873 and 1884 can very properly be classed under one of the following heads :

1st. That the first case or cases originated, de novo, in Austin ; the germ or micrococci or infectious matter springing into active work and extending its conquests by rapid invasions over the entire city.

2nd. That the first cases resulted from importing the active agent of the disease, either in the persons or by their clothing from infected districts.

There must have been a starting point, either in the air, soil, clothing or in persons.

It is a matter of grave importance to the city of Austin and to her future growth and prosperity to arrive at a fair and reasonable conclusion on these two points. If the *germs* of "dengue" hybernate in Austin, or develop from local conditions of air, soil or water, and make incursions at pleasure, the reputation of the city for healthfulness will be questionable. But if these germs are imported, then by quarantine and sanitation the disease may be kept out of the city or controlled by isolation if it enters it.

Did it originate here or was it imported ?

To disprove its origin, de novo, in Austin is to prove by its history and by the facts, its importation. If by importation, it must have been through the air carrying the germs, or by persons.

"Dengue" has prevailed in a large number of towns and cities in Texas during the last three months ; and at first thought it would appear reasonable to say, that it was owing to an epidemic condition of the atmosphere. I submit, however, this proposition, if such an atmospheric condition did exist was that condition *alone* sufficient to originate cases of dengue? It is believed, so far as I know, by all investigators, that dengue has its own specific germ, which operates in the human system in a specific manner and produces certain characteristic manifestations. If this statement is correct, it would be an unwarrantable assertion, to say that epidemic atmospheric influence *alone*, would or could incubate and develop the specific germs of dengue.

I have not been able to learn anything definite in reference to the origin of the epidemic in 1873. But I believe I have been able to find the first two cases that occurred in 1884. These cases were a Mr. Street and Mrs. Hall, who came from Galveston and Houston, where dengue was said to be prevailing. Soon after they came back to Austin they were attacked with dengue. The nurses and neighbors were soon victims to it and from these infected points the disease developed in several localities in the city. Now to say that the germs of the disease had been latent from 1873, for eleven years, would be too contrary to facts in its history to be entertained.

The history of epidemics of dengue all over the world are histories of importation, from place to place. The epidemic of 1824 and 1825, in the West Indies, was traced directly from Aden, in Arabia, and to Aden from Zanzibar, in Africa. Again, in 1827, the disease was epidemic in the West Indies, whence it spread to the United States, reaching New Orleans in the spring of 1828, and Savannah and Charleston in the summer and autumn of the same year. In 1870 and 1871 the disease was epidemic in Zanzibar, and was imported by the ship "Somalis" to Aden, in Arabia, and along the Arabian coast to Port Said and Cairo, in Egypt, following the lines of trade and commerce. Mooden, Sheriff of Madras, says: "The steamers 'Jumna' and 'Dalhousie' carried it from Aden to Bombay, and by the lines of railway all over India." Dr. W. H. Cock says: "An epidemic of dengue prevailed on the island of St. Bartholomew, West Indies. Three men went on the sloop 'Maxwell,' from St. Christopher to St. Bartholomew, and carried it back with them, and it spread from them all over the island."

Take the three main epidemics that have prevailed along the Southern coast, viz: 1828, 1850 and 1880, and it is believed the first cases were clearly traceable to importation. The epidemic of 1873 first prevailed in New Orleans, and was imported to Galveston, and spread over many parts of the State, following the lines of travel and commerce.

I might give in historic detail any number of instances showing that in epidemics of dengue the starting points—the commencement—the first cases—have been imported from infected localities. On the other hand, I find no historic instance in the United States where the dengue was traceable or believed to originate from local insanitary causes. It is believed by some persons that the turning up of the soil in the streets, in July and August, had much to do in producing the present epidemic. Whether it was a factor of any significance in producing a favorable atmospheric condition for the spread of the disease, I know not. No doubt, from a sanitary point of view, it was an ill-advised measure, and should not again be allowed during the hot months. But, in my opinion, it had nothing to do with *originating* the disease.

IT WAS IMPORTED.

The evidence, to substantiate this proposition, is, in my opinion, satisfactory. Upon careful investigation, I find the first case occurred on the 22nd day of July, on the block just west of the market house. Mrs. Emma Hood made a visit to Galveston on the 11th July, and remained in the city eight days. During her stay a Miss Tornblon, a member of the household where Mrs. Hood was boarding, was taken sick.

Mrs. Hood kindly ministered to her wants until her return to Austin, on the 20th of July. This young lady had the dengue fever, but neither Mrs. Hood nor her friends knew it at the time. On the 22nd of July—two days after returning to Austin—Mrs. Hood was taken down with a fever. I was called to see her, and found her suffering severely from what I supposed was a "bilious attack." I did not then recognize the disease as dengue.

But her subsequent symptoms and history for several days marked her case as one of dengue. Twelve other cases have occurred in her house, and she has nursed them, and has had no other symptoms of the disease. Her husband, Prof. Hood, and a young lady boarder, were taken down on the . th of August—twelve days from the attack of Mrs. Hood. From three to six days later, two other persons, who visited Mrs. Hood during her sickness, were taken with dengue. The disease spread then rapidly along West Hickory street, so that in a very few days almost every family for three blocks were attacked with it.

The next case was Miss Howard, who was taken sick on the 24th or 25th of July. Miss H. visited Houston on the 9th of July, and remained there seven days, attending a Baptist association. Miss H. was formerly from Galveston to Austin, and, naturally enough, she associated with her old friends from Galveston. She visited friends at Hempstead on her way to Austin, reaching here about the 23rd, when, in two days, she was taken with a fever, which kept her sick and prostrated for ten or twelve days. About one week after her recovery her mother was taken sick with what was then recognized as dengue. Miss H. has not had any other symptoms indicating an attack of dengue since her sickness in July. All her family and friends generally have had the disease. Miss H., so far as we know, was not in an infected district, but associated, intimately, with those from Galueston, where the disease did exist. It is known that clothing, merchandise, trunks, etc., etc., carry the infectious germs long distances. Is lt reasonable to conclude, from these facts, that Miss H. was infected in this way? I have no way of explanation, if this is not satisfactory. These being the first cases, became the starting points or infected centres from which the epidemic spread. What local atmospheric condition was present favoring and so rapidly spreading the disease I do not know. The prevailing winds were from the south and southeast; the weather dry and hot; the city at the time was quite healthy and free from epidemic diseases.

### SYMPTOMS.

Dengue is a disease of a variety of manifestations. In one case the burden of the disease is in one organ, and then in other cases the symptoms and sequelæ show that other and different organs are more seriously affected. *Fever*, ranging from 100 to 104 degrees, *pain* in the limbs, back or head, *loss* of appetite, and great *prostration* were the most constant symptoms. It is an eruptive disease, the eruption in some form, and at some stage of the disease, showing itself in probably 90 per cent. of the cases. The initial eruption was usually of a scarlatinal or erythematous rash on the face neck or chest. The secondary, or terminal rash, was more constant, occurring after defervescence, from the fifth to the twenty-fourth day. This rash sometimes resembled measles, urticaria, or herpes, but more commonly the rash was of a miliary form. Of all the cases where I made a careful and successive examination, the rash, in some variety, was found in about 80 per cent. of the cases. Stiffness in the back of the neck, rheumatic pains, and swelling of the joints, and a feeling like the joints were being pulled apart, were frequent and distressing symptoms. Other cases, deep aching and throbbing of the temples and balls of the eyes. Then, again the gastric and enteric symptoms were most distressing, as vomiting and irritable stomach, even to the throwing up of black vomit, (as in two marked cases).

Again, inflammation of the descending colon, about the sigmoid flexure, with dysentery, were the leading and distressing symptoms, usually after the acute stage had passed. In some, aching and throbbing over the region of the kidneys with more or less suppression of urine, others, a mental dulness and depression of spirits with a feeling of faintness, even when lying down, indicating a weak and troubled heart. Then in others a distressing and unaccountable prostration, out of all proportion to the severity of the attack. The truth is, the functions of all the organs were more or less deranged. The blood was poisoned and changed in its elements by the introduction into the body, of the infectious material, be that material germs or micrococci, the results were the same.

The epidemic has not been a mild form of the disease ;on the contrary it has been a severe and serious one. While but few deaths are recorded as resulting directly from dengue, (probably not over ten or twelve deaths in Austin), many persons will suffer from the sequelæ for months to come, and will date back their sickness, and it may be in many cases their deaths, from the dengue of 1885.

In the deaths recorded as having resulted from dengue, at least in two cases, the kidneys were the organs more seriously affected. In one case the death occurred through affection of the brain. In one the stomach, with black vomit. In one—a small infant—through the heart, and in one man, the nervous system gave down, as from a blood poison.

Of the deaths resulting from dengue, only one post-mortem was held, so far as I know. This case is of such interest I will briefly relate it.

James Artforth, a german eight months in the United States, aged 23, with history of good health previous to the attack of dengue. On the 22nd of September he was taken down with dengue which lasted him for several days. No physician attended him. He began a slow convalescence, until October 2nd he had evidently a relapse, with high fever, pain in the side and back, with distressed breathing. This condition continued until the 5th of October, when seeing he was very sick I sent him to the hospital. He had a high fever, temperature 104. Physical signs of pneumonia, but neither cough nor expectoration. Heart's action weak, face white and anxious, and great prostration. These symptoms continued with little variation, except a gradual and increasing prostration, until the 10th of October when he died.

A post-mortem was held sixteen hours after death. Present at the examination, Drs. J. W. and Frank McLaughlin, R. S. Graves and W. J. Burt. Dr. Frank McLaughlin made the autopsy. There was nothing to attract attention from inspecting the body. After exposing the organs by the usual incisions, the pericardium was opened and the heart removed and examined.

The right auricle and ventricle were very thin and flabby; The left ventricle enlarged and its walls considerably thickened. On opening the cavities, they contained a small amount of bloody water but no clots; valves seemed sound.

*Lungs*—Slight pleuritic attachments to the left lung, and very strong adhesions to the right lung, particularly to the diaphragm. Both lungs had a reddish aspect, with grayish or ash colored spots here and there over the surface. By making incisions in the lungs in various directions, the cut surfaces had the appearance of red hepatization, with a reddish, frothy fluid oozing from the cut surfaces. The lungs and parts of them floated in water. No pus was found. A thin, watery, bloody fluid issued from the cut blood vessels.

*Liver*—was large—particularly the right lobe; it had yellowish instead of a brownish appearance, externally. When cut into it was a fatty liver, and an oily, red tinged fluid was easily removed with the scalpel. Dr. J. W. McLaughlin examined parts of it under the microscope and found its composition largely to be fat cells, and the whole field covered with the black-headed micrococci. White paper was stained with bile when pressed to the cut surfaces, in fact the liver seemed full of bile in all its parts.

*Spleen*—was enlarged, but no special changes observed in it.

*Kidneys*—The cortical portion was congested, the pyramids swollen and inflamed in the lower and middle portion of the kidneys, but tough and white in the upper third. The kidneys through both structures were hard in cutting in a longitudinal direction.

*Blood.*—No c'ots were found in the body, the blood was very thin and watery and seemed to be disorganized. When thrown on a white cloth it merely made a stain like bloody water.

How much of these pathological changes observed are the result of dengue, can only be conjectural. Future autopsies may confirm them. It is to be regretted that so little attention has been given this matter in other epidemics throughout the southern coast States. I have not been able to find a history of a post-mortem held on a person who had died of dengue, through all these epidemics. Whether the microscope will give us a more correct knowledge of the disease remains to be seen. If my distinguished friend, Dr. J. W. McLaughlin, is able to isolate the micrococci, he has discovered in dengue patients and to propagate them through two or more generations and then produce dengue by introducing them into the human body, he will stand with Koch and Pasteur as a discoverer, and his name, like that of Jenner, will go sounding down through the generations as a benefactor of his race. Let us hope that the Dr. will find the living germ that produces dengue, and he or some other one find the germicide, which will cut off its invasions and inhibit its epidemic work.

### TREATMENT.

Dengue being a self-limited disease, disposed to a favorable termination, the large majority of cases require very little medical treatment. However as the disease is not so simple in its ultimate results, the vigilance of the medical attendant should not be relaxed, but he should carefully watch for complications that so often arise, and treat them promptly. In a large percent of cases the patient needs to be placed in bed, and a warm foot bath and warm drinks administered, with cold applications to the forehead and eyes. A quieting mixture as a Dover's powder or a bromide of potash, morphia and aconite mixture should be given. A mild purgative is often required. If the pains are severe and the temperature runs high then the salicylate of soda in 10 to 20 grains, every few hours usually gives satisfactory relief. There is no specific for the disease yet discovered. It must be treated expectantly and conservatively, meeting complications as they arise, supporting the weak organs and promoting functional action of the various secreting organs, hoping in this way to more rapidly eliminate the poison from the blood and bring about a restoration to health.

